# Unobtrusive Monitoring the Daily Activity Routine of Elderly People Living Alone, with Low-Cost Binary Sensors

**DOI:** 10.3390/s19102264

**Published:** 2019-05-16

**Authors:** Ioan Susnea, Luminita Dumitriu, Mihai Talmaciu, Emilia Pecheanu, Dan Munteanu

**Affiliations:** 1 Department of Computer and Information Technology, University “Dunarea de Jos” of Galati, 800146 Galați, Romania; Luminita.Dumitriu@ugal.ro (L.D.); Emilia.Pecheanu@ugal.ro (E.P.); Dan.Munteanu@ugal.ro (D.M.); 2 Department of Mathematics and Informatics, University “Vasile Alecsandri” of Bacau, 600115 Bacău, Romania; mtalmaciu@ub.ro

**Keywords:** long-term activity monitoring, anomaly detection, modeling the living space, virtual pheromones

## Abstract

Most expert projections indicate that in 2030, there will be over one billion people aged 60 or over. The vast majority of them prefer to spend their last years at home, and almost a third of them live alone. This creates a growing need for technology-based solutions capable of helping older people to live independently in their places. Despite the wealth of solutions proposed for this general problem, there are very few support systems that can be reproduced on a larger scale. In this study, we propose a method to monitor the activity of the elderly living alone and detect deviations from the previous activity patterns based on the idea that the residential living environment can be modeled as a collection of behaviorally significant places located arbitrarily in a generic space. Then we use virtual pheromones—a concept defined in our previous work—to create images of the pheromone distribution maps, which describe the spatiotemporal evolution of the interactions between the user and the environment. We propose a method to detect deviations from the activity routines based on a simple statistical analysis of the resulting images. By applying this method on two public activity recognition datasets, we found that the system is capable of detecting both singular deviations and slow-deviating trends from the previous activity routine of the monitored persons.

## 1. Introduction

According to the projections of the United Nations Organization [1], by 2030, the number of older persons (60 y.o. or older) will exceed 16% of the total world population. That is over one billion people. A large proportion of these people (32% in Europe [2]) live alone. Despite the obvious difficulties of living alone, 90% of older adults still prefer to spend their last years in the comfort of their homes [3] rather than going to a nursing home.

At the social scale, the economic impact of the population aging goes far beyond the significant increase of public expenditures on age-related health issues and influences the labor market, migration, and eventually the global economic growth [4,5].

At the individual and family scale, as the professional caregivers become a scarce and expensive resource, the burden of care for the elderly affected by debilitating diseases rests on the families and a small number of volunteers. This often results in even higher costs, because one third of these caregivers are affected by depression and other psychological disorders [6].

On the other hand, the unprecedented progress of technology (wearable sensors, ICT, smart phones and other mobile devices, wireless communications, etc.) enables the development of effective solutions to help older people to live independently in their homes.

The research aimed at harmonizing the aging society with the technological innovations received a variety of names in the literature. For example, the term “smart home” designates a residence augmented with sensors, actuators, data processing, and communication devices aiming to improve comfort, security, the healthcare of the inhabitants, or to contribute to a better energy management of the building [7,8].

Telemedicine, telemonitoring, and telecare are partly overlapping concepts designating the technologies capable of providing remote medical consultations, remote monitoring of certain physiological parameters, remote supervision of the medical treatments, or other health-related activities or habits [9,10].

Ambient assisted living (AAL) designates the use of any technological means, integrated in the living and working environment, in order to enable users to live independently and to remain active while avoiding social isolation into old age [11,12].

Gerontechnology [13,14] is concerned with the research on the specific medical and social needs of the elderly, and the technological response to these needs, not necessarily in connection with the living environment.

The definition of “e-Health” varies from “the integration of the Internet into health care”, to “the use of ICT in the health care sector for clinical, educational, or administrative purposes” [15].

**Figure 1:**
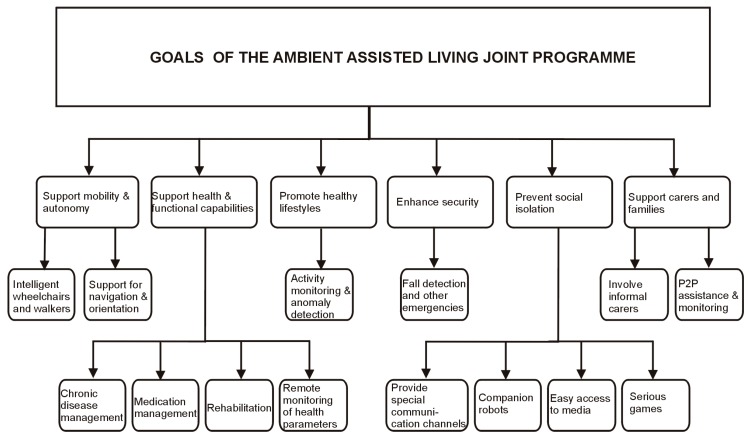
A taxonomy of the research directions in ambient assisted living (AAL) based on the objectives formulated by the AAL Joint Programme.

This multitude of perspectives led to a wealth of solutions. Starting from the comprehensive surveys of the recent literature available in Refs. [7,8,16,17,18,19,20], we derived a taxonomy of the research directions in AAL based on the explicit goals of the AAL Joint Programme, formulated in Ref. [21]. It is shown in Figure 1.

**Figure 2:**
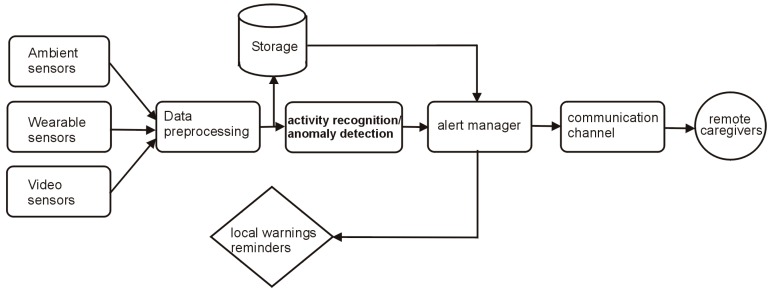
A general structure of a typical AAL system.

The general structure of a typical AAL system is presented in Figure 2. Basically, such a system includes a number of sensors to track the interactions between the assisted person and the environment and uses the sensor data to infer information about the activities of the subject. Furthermore, the activities are analyzed from the perspective of the health and safety of the assisted person and, upon detection of any anomaly or risk, the system either issues local warnings/reminders or sends alert messages to remote caregivers.

An excellent review of the ambient sensors used for elderly care is available in Ref. [22]. This class of sensors includes: Passive infrared (PIR) motion detectors, magnetic switches, pressure, temperature and CO_2_ concentration sensors, RFID (radio frequency identification), sound and vibration sensors, and others based on emerging technologies such as silicon photomultipliers (SiPMs) [23].

A special type of ambient sensors are the video sensors. The research on video-based activity recognition evolved independently from AAL, in the larger context of machine vision, with notable applications in surveillance, health monitoring, and general human–computer interfaces [24,25]. As shown in Ref. [25], video-based human activity recognition delivered the most promising results, but this approach remains the most intrusive and is considered inappropriate for long-term monitoring.

Finally, wearable sensors [26,27] are designed to be worn 24/7 and constantly monitor certain biomedical parameters (e.g., pulse rate, blood pressure) or the physical motion of the subjects (e.g., the gait or falls). Notable examples of such sensors are inertial sensors (accelerometers and gyroscopes), sensors for peripheral capillary oxygen saturation (SpO_2_) or galvanic skin response (GSR). In most cases, they are attached to a wristband or included in smart watches or smart phones.

The complexity of the data preprocessing subsystem is closely linked to sensor technology. For example, binary sensors need only minimal preprocessing (e.g., pulse counting), but others, like the SpO_2_, GSR, inertial, and video sensors, require specific and relatively complex signal conditioning and calibration modules.

In what concerns the implementation of the actual data processing subsystem, this largely depends on the granularity of the desired output: While most of the proposed solutions attempt a fine grained discrimination of the activities of the daily life (ADL) of the assisted persons [28,29], others are focused on long-term lifestyle monitoring and on detecting deviations from certain expected behaviors considered “normal” [30,31].

Despite the remarkable performances recorded in the laboratory and reported in the literature, there are still very few real-life applications of activity recognition to support the independent living of the elderly. Among the reasons for this apparent paradox, we count:

- The set of sensors used and their spatial distribution is highly dependent on the specific needs of the assisted person and their living environment, hence the difficulties to reproduce the solution in a different context. Often, even a simple sensor change may result in the need to train the network again. In other words, these systems are not scalable and have low tolerance to sensor faults.
- Most of the existing solutions are perceived as intrusive and raise privacy concerns at the users.
- They are expensive.
- Finally, it is not without importance to note that most of the existing solutions for AAL tend to treat the assisted persons as totally helpless and neglect the fact that many of them are capable and willing to provide some sort of assistance to similar peers. Knowing that assigning even very limited responsibilities to the elderly, like watering a plant, can help them to live happier and longer [32], we suggest that involving the assisted persons in ICT-mediated groups for P2P health and lifestyle monitoring might be psychologically beneficial for them.

In this general context, the study described in this article aims to overcome some of the abovementioned limitations. We propose a non-intrusive data-driven solution to detect deviations from the long-term daily activity routine of the elderly people living alone based on low-cost binary sensors, like PIR motion detectors or magnetic door contacts. Our research hypothesis can be formulated as follows:

*“The structure of the activities can be encoded in a series of visual representations of the interactions between the user and their living space,* *starting from the data provided by a set of low-cost binary sensors, and this encoding is sufficient for detecting anomalies in the daily activity routines.”*

Key contributions:

Starting from the distinction formulated in Ref. [33] between “location” (defined as a specific position in space) and “place” (a location with behavioral meaning attached), we propose an abstraction of the residential living space represented as a collection of behaviorally significant places (bedroom, kitchen, bathroom, living room), located arbitrarily in a generic Cartesian space. A number of low-cost binary sensors (PIR motion detectors and magnetic door contacts) are located in each place of this space. Furthermore, we use the sensor data and the concept of “virtual pheromones”—defined in our previous work [34] as “traces created by the agents not in the environment, but in a representation thereof, a map”—to create visual images of the pheromone distribution maps, which describe the spatiotemporal evolution of the interactions between the user and the environment. We propose a method to detect deviations from the activity routines by computing a similarity index between these pheromone-based activity maps and a set of reference images created starting from the average values of the sensor data. This data-driven approach does not require explicit labelling of activities for detecting deviations from the previously recorded routine.

The remainder of this presentation is structured as follows:

Section 2 is a brief review of the closely related work. Section 3 contains a description of the proposed method. In Section 4, we present the experimental results, while Section 5 is reserved for discussion.

## 2. Related Work

According to Ref. [35], there are four types of abnormal (or deviating) behaviors detectable starting from sensor data:

- Known behavior in a deviating spatial context (e.g., sleeping in the kitchen);
- Know behavior occurring at a deviating moment in time (e.g., having dinner very late in the night);
- Known behavior with an abnormal duration (e.g., sleeping until noon, or spending too much time in the bathroom);
- Behavior resulting in abnormal/unexpected sensor firings patterns (e.g., abnormal gait or falling).

One particular type of abnormal behavior—the fall—has been extensively studied, and there exists a whole literature dedicated to fall detection systems (see Refs. [36,37]).

The systems for detecting all the other types of abnormal behavior (surveyed in Refs. [12,18,29,31,38,39]) implicitly rely of some sort of synthetic representation of the human activity in a spatiotemporal context.

**Figure 3:**
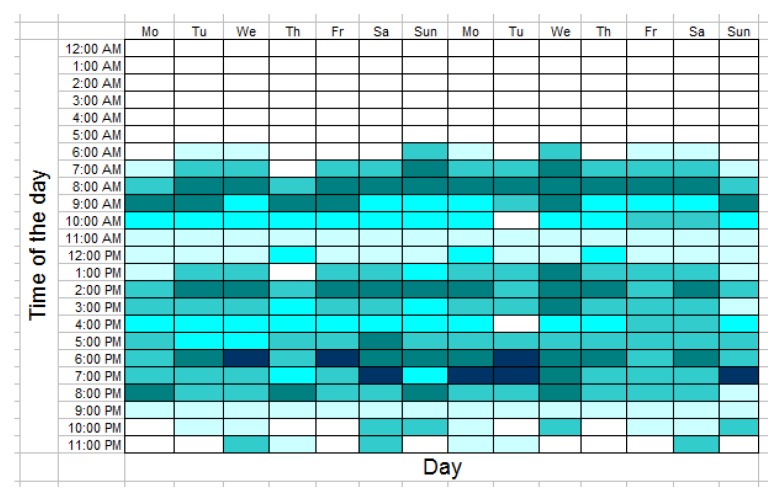
An example of color-coded activity density map.

For example, Ref. [40] proposed color-coded “activity density maps”, wherein black cells represent time away from home, white corresponds to very low densities of activity, and color shades between yellow and dark blue encode the number of sensor events per hour. A similar solution but without detecting the time away from home intervals and with different color codes can be found in Ref. [41] (see Figure 3).

It should be noted that this type of activity maps does not embed any information about the space where the activities occur. However, the spatial dimension of the activities remains important from the perspective of detecting deviating behaviors. The authors of Refs. [42,43] solved the problem of linking sensor data with the locations where the observed activities occur using the concept of virtual pheromones defined in Ref. [34].

Considering a metric space (*M*,*d*), where *M* is a set of points and *d* is a distance function, we defined a “virtual pheromone source” *S* as a point, characterized by a position and a real positive scalar *P*, called “pheromone intensity”. The pheromones *diffuse* in space, so that at the distance *x* from the source, the pheromones from source *S* can be “sensed” with the intensity *p*(*x*)*:*

(1) $$p(x)=\left\{ \begin{array}{ll} P\left(1-\frac{x}{\sigma }\right) & for\;x\leq \sigma \\ 0 & for\;x>\sigma \end{array}\right.,$$

where σ is a positive constant called diffusion range. If *N* discrete pheromone sources exist, then the aggregated resultant intensity due to the *superposition* of effects is *P_R_*:

(2) $$P_{R}=\sum_{k=1}^{N}P_{k}\left(1-\frac{d_{k}}{\sigma }\right),$$

where *P_k_* is the intensity of the source *S_k_* and *d_k_* is the distance from the current point to the source *S_k_.*

Finally, the virtual pheromones *evaporate*, i.e., the intensity of the pheromone sources decrease with time. Assuming a linear variation:

(3) $$P_{R}=\sum_{k=1}^{N}P_{k}\left(1-\frac{d_{k}}{\sigma }\right)\left(1-\frac{t-t_{k}}{\tau }\right),$$

where *t_k_* is the moment of creation of the source *S_k_*, and τ is the evaporation constant.

**Figure 4:**
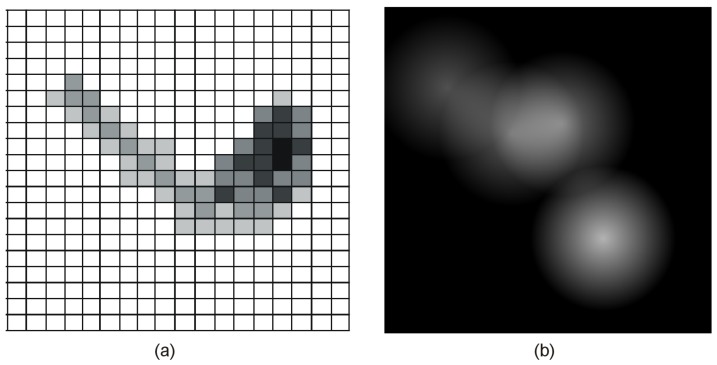
Examples of pheromone-based activity maps described in Refs. [42,43].

Starting from this model, in Ref. [42], it was assumed that there exists an indoor localization system (e.g., the one presented in Ref. [44]) capable of providing accurate coordinates of the assisted person within a known environment. Periodically, a marking subsystem reads the location of the user and creates pheromone marks in the corresponding position of a map of the environment, conveniently structured as a grid of cells. The local pheromone marks diffuse in space and evaporate in time, resulting in an aggregated track that describes the recent motion of the observed subject, as shown in Figure 4a. Subsequently, the aggregated track is cellwise compared for similarity with a reference track generated in the same manner for a period of time selected by a human operator. Dissimilarities that exceed a certain threshold are reported. The authors argue that this system has good performances with respect to localization errors, but in our opinion, it still remains very sensitive to even small variations in the environment (e.g., moving a piece of furniture may lead to changes of the user's habitual motion pathways through the environment, resulting in large deviations from the recorded routine and systematic false alerts).

The solution described in Ref. [43] does not need a localization subsystem: The virtual pheromone sources are placed on a Cartesian map of the environment in fixed positions, each corresponding to a binary sensor. Every time the sensor is triggered by the presence of the user in its proximity, the pheromone source located in the corresponding position on the map releases a fixed amount of pheromones. Figure 4b shows the pheromone intensities map generated by four sensors recently activated, in the assumption of a linear diffusion. The pheromone intensities are encoded in shades of gray, between black (no pheromones detected in that point) and white (maximum intensity of pheromones in the respective position).

Images of the type presented in Figure 4b can be considered “snapshots” of the recent activity within the observed area. Pairs of such images (*x*,*y*) can be compared for similarity using the structural similarity index SSIM defined in Ref. [45].

(4) $$SSIM(x,y)=\frac{\left(2\mu_{x}\mu_{y}+C_{1})(2\sigma_{xy}+C_{2}\right)}{\left(\mu_{x}{}^{2}+\mu_{y}{}^{2}+C_{1})(\sigma_{x}{}^{2}+\sigma_{y}{}^{2}+C_{2}\right)},$$

where $\mu_{x},\mu_{y},\sigma_{x}{}^{2},\sigma_{y}{}^{2}$ are the mean and variance values and $\sigma_{xy}$ is the covariance for x and y. *C_1_* and *C_2_* are notations for:

(5) $$C_{1}={\left(k_{1}L\right)}^{2}\;;\;C_{2}={\left(k_{2}L\right)}^{2},$$

with $k_{1},k_{2}<<1$, and *L* is the dynamic range of pixel values. Default values (in MATLAB) for $k_{1}$ and $k_{2}$ are 0.01 and 0.03, respectively, and *L* = 255 for 8-bit grayscale images.

The authors of Ref. [38] created pheromone map images every 10 min for 10 weeks and computed the similarity index of the images for the corresponding time windows in different weeks. The resulting similarity matrix was analyzed by human experts in order to identify deviations from the ADL routine. In the absence of a method to average series of images, it was not possible to define a reference of “normal” activity levels for comparisons.

The studies presented in Refs. [42,43] have the merit that they avoid the difficult problem of the explicit modeling of the activities but remain sensitive to small variations in the environment and to sensor faults. The solution described here attempts to overcome both these limitations.

## 3. Method and Datasets

Arguably, from the perspective of assessing the daily life routine, many details concerning the environment where the elderly live (e.g., the surface of the apartment, the type and spatial distribution of the furniture, the size and location of the appliances) are unimportant. Stripping away all these environmental details would greatly simplify the task of long-term monitoring of the activity of the people living in the respective environments.

**Figure 5:**
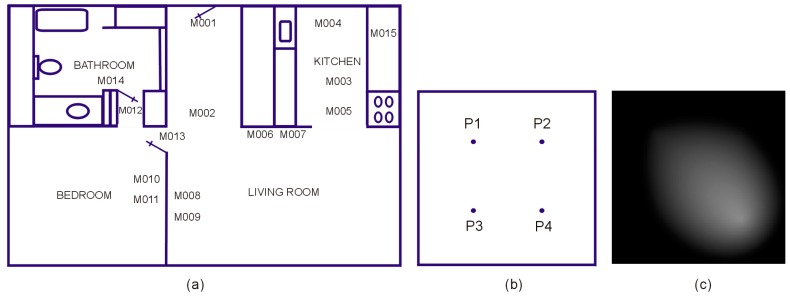
(**a**) Floor plan and sensor layout for CASAS HH126 testbed, (**b**) the corresponding generic space, and (**c**) a sample pheromone map describing the activity within this space.

To this purpose, we propose an abstract model of the living environment consisting of a set of behaviorally significant “places” arbitrarily distributed in a generic space. For example, Figure 5a presents the floor plan and sensor deployment for one of the CASAS smart homes testbeds (namely, HH126, see Ref. [46]), while Figure 5b,c shows the corresponding generic space with the location of the four places (P1-bedroom, P2-Kitchen, P3-bathroom, P4-living room) and a sample pheromone distribution map for this space.

For this particular sensor layout, the correspondence between sensors and places is:

(6) $$\begin{array}{l} \left\{P_{1}\}=\{M010\}\cup \{M011\}\cup \{M013\right\}\\ \left\{P_{2}\}=\{M003\}\cup \{M004\}\cup \{M005\}\cup \{M015\right\}\\ \left\{P_{3}\}=\{M012\}\cup \{M014\right\}\\ \left\{P_{4}\}=\{M001\}\cup \{M002\}\cup \{M006\}\cup \{M007\}\cup \{M008\}\cup \{M009\right\} \end{array},$$

where $\left\{P_{i}\right\}$ denotes the set of sensor events associated with the respective place, Mk is the motion detector sensor *k*, and {Mk} is the set of events generated by the sensor Mk.

**Figure 6:**
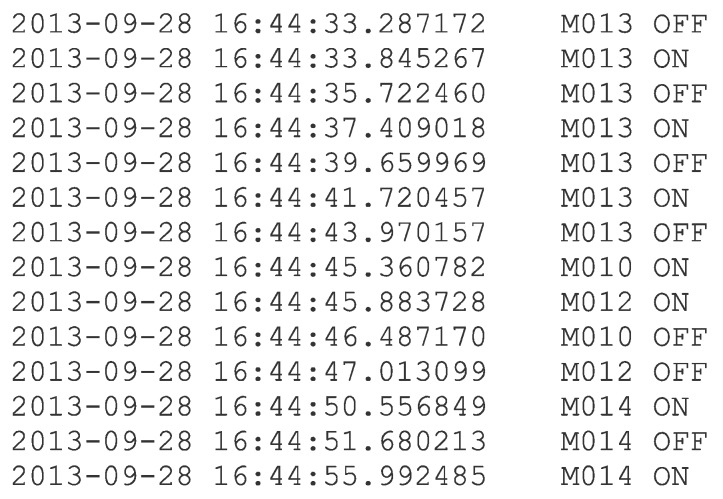
The raw data format in the CASAS datasets.

The raw sensor data for the CASAS datasets, downloaded from Ref. [47], is structured as shown in Figure 6.

**Figure 7:**
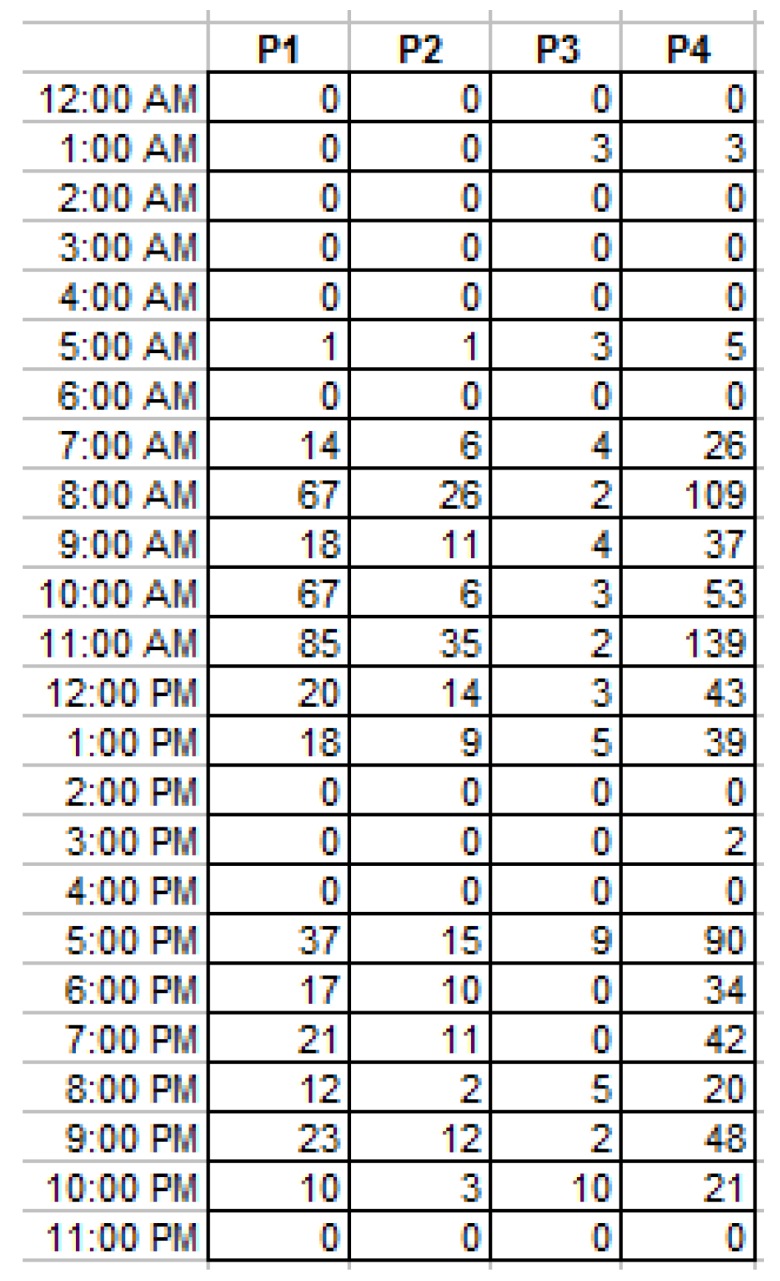
The synthetic activity data for one day after the preprocessing phase.

In the preprocessing phase, the following operations were performed on the raw data:

- All the OFF events associated with the motion detectors were filtered out, because these sensors are designed to turn OFF automatically a few seconds after the moment they are triggered, regardless of the external activity.
- Repeated signals from the same sensor occurring faster that one event per minute were also filtered out as irrelevant.
- The sensors were associated with the places P1–P4 according to the rules in Equation (6).
- Finally, the sets of events $\left\{P_{1}\}....\{P_{4}\right\}$ were sorted by time intervals of one hour each, and the cardinals of these subsets were presented in distinct daily activity files, as shown in Figure 7.

**Figure 8:**
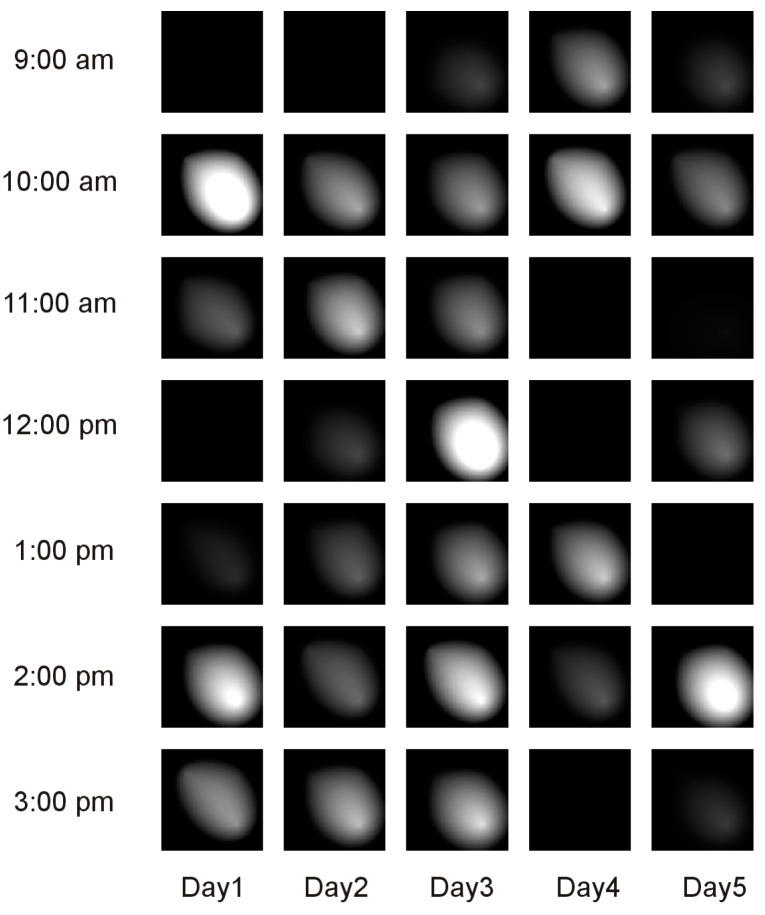
A fragment of the activity map built with hourly images of the pheromone distributions.

Using these data and the concept of virtual pheromones modeled with Equation (3), we created for each day a set of 24 images (128 × 128 pixels in size) representing the hourly pheromone maps. A fragment of the resulting activity maps for five days is shown in Figure 8.

Compared to the previously described activity maps, like the one depicted in Figure 3, this type of activity map is substantially richer in information because it contains not just an overall index of the intensity of activity but also embeds information about the places where the activities occurred and the relative weights of these places from the perspective of the spatial distribution of activity.

The corresponding hourly images can be compared for any two days, as in Ref. [43], but with little practical benefits. A more efficient way to use these maps is by creating a set of reference images starting from the average values of sensor data over a certain time interval. The reference time interval (e.g., a week) can be manually selected by a caregiver or a member of the family, as in Ref. [42], but it is also possible to select a number of consecutive days with the lowest dispersion of the values of sensor data.

In the first stage of our experiment, we created the reference images by averaging the sensor data hour by hour for an arbitrary interval of one week. Then, we computed with Equation (4) the structural similarity indices for the entire interval studied with respect to the reference image set.

**Figure 9:**
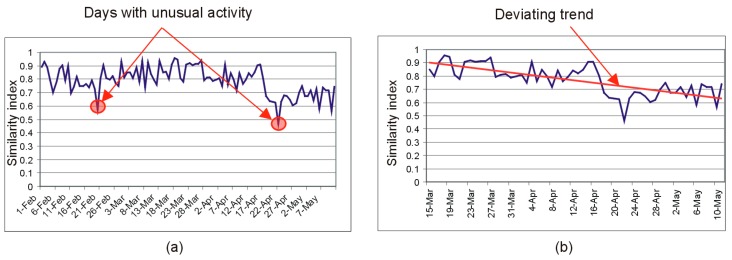
(**a**) Days with a low similarity index may indicate unusual activity. (**b**) Descending trends in the evolution of the similarity index may indicate deviations from the activity routine.

By simply plotting the similarity index against time, it is possible to identify either certain days with unusual activity (i.e., with lower similarity indices), as shown in Figure 9a, or trends of evolution that indicate deviations from the previous activity routine (Figure 9b).

The CASAS HH126 dataset is not annotated, but it has a useful property: The set of sensors was upgraded after the data recording started. Specifically, on 18 April 2014, new sensors (M002, M003, M004, M005, M006, M008, M010, M014 and M015) were added to the initial set. This major shift in the sensor data flow should be clearly identified by the activity monitoring system as an event of the type illustrated in Figure 9b. To make use of this property, we selected the time interval for this study for 60 days between 1 April and 30 May 2014 to include the date of 18 April 2018.

Further, it is worth noting that the CASAS HH126 testbed contains exclusively PIR motion detectors and magnetic door contacts as sensors—the same type of sensors that are extensively used and already present in millions of homes throughout the world as components of low-cost intrusion security systems. Demonstrating that this type of sensors can be used for efficient long-term activity monitoring may stimulate the manufacturers of security systems to implement certain functions of activity monitoring as special features of their products.

In order to demonstrate that the proposed solution is also capable of detecting isolated days with unusual activity of the type illustrated in Figure 9a, we repeated the experiment on a smaller (18 days between 20 November 2008 and 7 December 2008) but fully annotated dataset, namely Kasteren House C, downloaded from Ref. [48]. The Kasteren House C is a two story building, with two bedrooms, two bathrooms, and multiple other living spaces, but it easily fits into the four places model of the living environment, by using the correspondence between the activity relevant places and the sensors defined in Equation (7):

(7) $$\begin{array}{l} \left\{P_{1}\}=\{S05\}\cup \{S29\}\cup \{S39\right\}\\ \left\{P_{2}\}=\{S07\}\cup \{S13\}\cup \{S18\}\cup \{S20\}\cup \{S21\}\cup \{S22\}\cup \{S23\}\cup \{S27\}\cup \{S30\right\}\\ \left\{P_{3}\}=\{S08\}\cup \{S10\}\cup \{S11\}\cup \{S16\}\cup \{S25\}\cup \{S35\}\cup \{S38\right\}\\ \left\{P_{4}\}=\{S06\}\cup \{S15\}\cup \{S28\}\cup \{S36\right\} \end{array}$$

where *Sxx* denotes the sensor with the *ID* == *xx.*

The preprocessing of this dataset was slightly different because the respective testbed contains three pressure sensors, placed under the beds and couch, which report unusually high levels of activity when the user was actually asleep or resting. Therefore, these sensors were conventionally muted for 10 min after each accounted event.

The availability of annotations allowed us to make a more accurate selection of the reference interval. To this purpose, we have used the minimum standard deviations of the duration of sleep over seven consecutive days as a measure of the uniformity of the lifestyle. With this criterion, we selected the week from 24–30 November 2008 as a reference interval for the Kasteren C dataset.

## 4. Experimental Results

### 4.1. Results with CASAS HH126 Dataset

**Figure 10:**
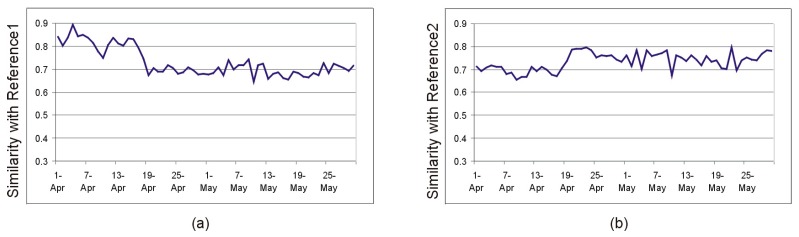
(**a**) The similarity index after comparison with a reference interval before 18 April 2014. (**b**) The similarity index after comparison with a reference interval after 18 April 2014.

In order to illustrate the change in the sensor data flow caused by adding new sensors on 18 April, we compared the similarity of the pheromone based activity maps for the interval 1 April 2014–30 May 2014 with two reference intervals, before and after the event of interest: Reference interval 1 (1–7 March 2014), and reference interval 2 (1–7 June 2014). The results are shown in Figure 10.

**Figure 11:**
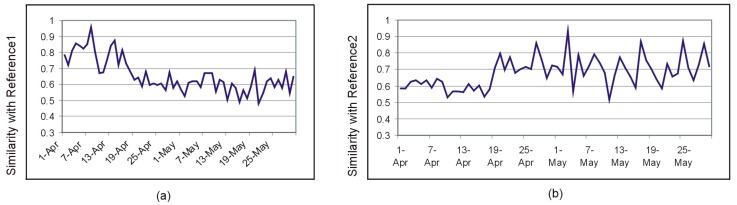
(**a**) The similarity index for the morning hours after comparison with a reference interval before 18 April 2014. (**b**) The similarity index for the morning hours after comparison with a reference interval after 18 April 2014.

Since many of the newly added sensors were located in the kitchen, we presumed that the dissimilarities between the intervals before and after 18 April are even more visible in the morning hours, when the user is likely to use the kitchen to prepare breakfast. The similarity index computed for just 3 h (7–10 a.m.) shown in Figure 11 supports this hypothesis.

**Figure 12:**
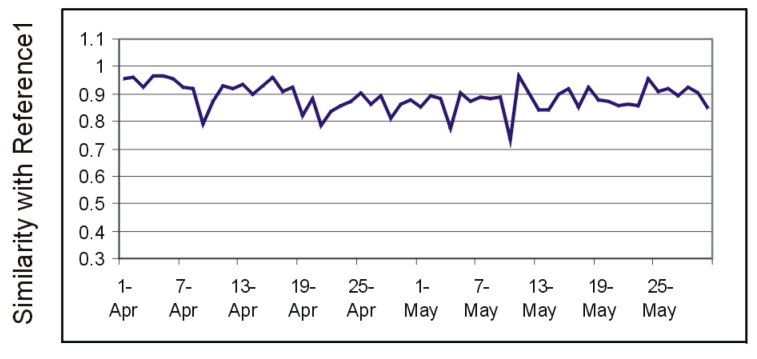
The similarity index for the night time (between midnight and 6 am) barely reflects the sensor change from April 18.

The bedroom was less affected by the sensor change in April 18—only one sensor (M10) was added—and therefore, the activity maps for the night time (between midnight and 6 am) barely reflect the moment of April 18 (see Figure 12).

**Figure 13:**
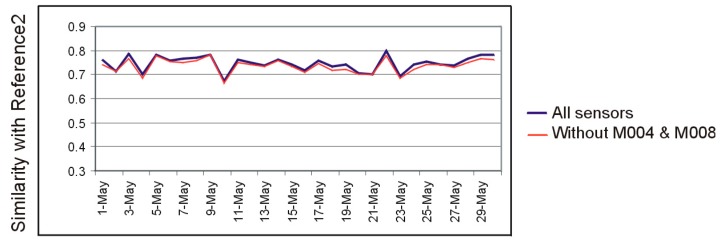
The results of a simulated sensor fault for M004 and M008 versus the results obtained with the original dataset.

This suggests that the relative influence of individual sensors on the overall capacity of the system to detect deviations from the activity routine is small, provided that there exists a certain level of redundancy in the sensing system. To verify this hypothesis, we simulated a sensor fault for M004 and M008 by filtering out the data provided by these sensors and compared the similarity index with that obtained with the original dataset. The result is shown in Figure 13.

### 4.2. Results with Kasteren House C Dataset

**Figure 14:**
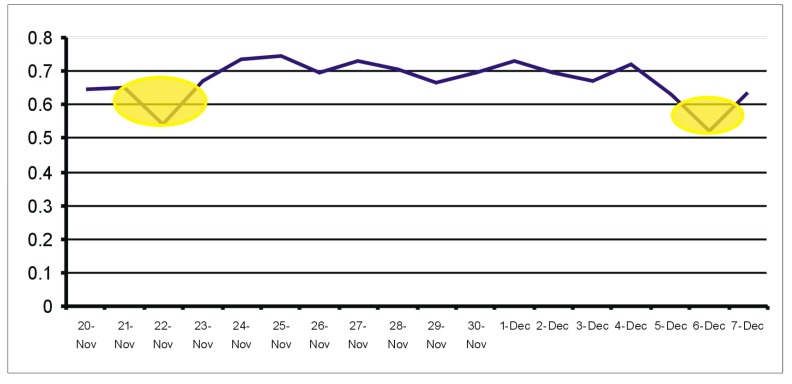
Days with dissimilarities from the activity routine in the Kasperen dataset.

Figure 14 shows the evolution of the similarity index computed for the entire Kasteren House C dataset versus the reference week 24–30 November 2008.

**Figure 15:**
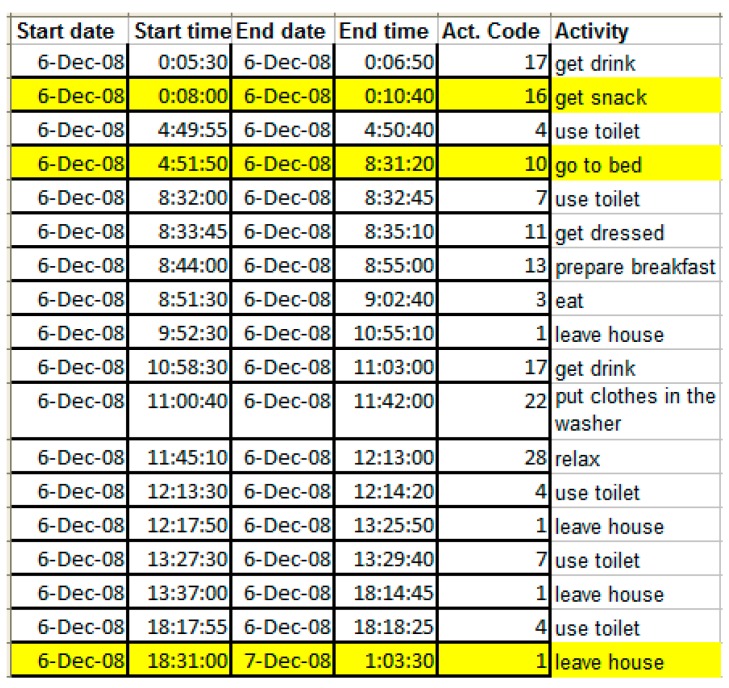
The activities from one of the days in the Kasteren C dataset, extracted from the annotations. Yellow highlights indicate unusual times for the respective activities.

The graph in Figure 14 indicates the dates of 21 and 22 November, and 5 and 6 December as days with unusual activity. Indeed, the activity list extracted from annotations (see Figure 15) reveals obviously unusual sleep and eating times in these days, or unusual times to leave the house and return.

## 5. Discussion

We presented a method to monitor the activity and detect deviations from the long-term activity routine of the elderly people living alone using low-cost, unobtrusive binary sensors. In our approach, the residential living space was reduced to a set of behaviorally significant places, located arbitrarily in a generic space. Each of these places was equipped with a number of sensors capable of detecting the presence of the assisted person. When triggered, the sensors activate a corresponding source of virtual pheromones located in the respective place. The pheromones diffuse in space and evaporate with time, and their density distribution maps encode the spatiotemporal evolution of the interactions between the user and the environment. Series of images representing pheromone-based activity maps were compared for similarity with a reference set of images created starting from the average values of sensor data over a certain time interval. By applying this method on two public datasets, we demonstrated that it is capable of identifying either singular days with unusual activity, or trends of evolution indicating deviations from the previous activity routine.

The proposed solution addresses all the major drawbacks of the typical long-term activity monitoring systems:

- It is based on low-cost PIR motion detectors and magnetic door contacts, which are totally unobtrusive and require minimal preprocessing;
- It is cheap;
- It does not need complex personalization and training;
- It is independent from the particular details of the monitored living environment (surface of the apartment, number and relative position of the rooms, size and location of the furniture, etc.);
- Provided that there exists a certain level of redundancy in the set of sensors, the system is tolerant to sensor faults.

The main limitation of the proposed method is that it is applicable strictly to monitoring people living alone.

**Figure 16:**
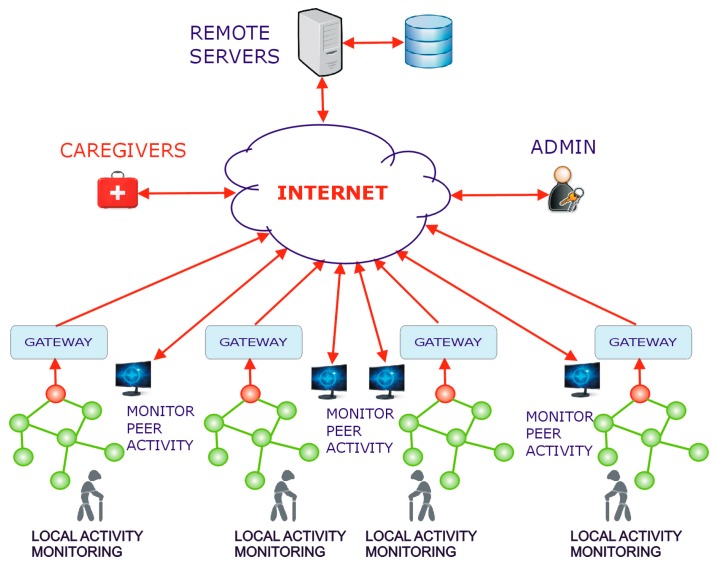
The structure of a network of individual monitoring systems that allows peer to peer monitoring.

It should be noted that is relatively easy to design a network of such residential activity monitoring systems that allows peer to peer monitoring of the activity routines. For example, in a network with the structure shown in Figure 16, the users may choose to (anonymously) share the output of their activity monitoring systems with a set of selected peers. The loss of privacy is minimal, but the psychological benefits of this mutual monitoring may be significant.

For the above-listed reasons, we believe that this type of long-term activity monitoring systems, completed with a low-cost, accelerometer-based fall detection device, and a panic button may indeed be applicable on a large scale, with reasonable economic costs.

Further research is required to clarify whether the proposed method is capable of comparing patterns of activity from different users. Our preliminary investigation suggests that this could be possible, provided that the users under comparison are monitored with similar sets of sensors. In principle, it is also possible to create rule-based “ideal lifestyle patterns” to be used as a reference for multiple users, e.g., for rehabilitation purposes.
